# Expanded dengue syndrome: subacute thyroiditis and intracerebral hemorrhage

**DOI:** 10.1186/1471-2334-12-240

**Published:** 2012-10-03

**Authors:** Muhammad Zaman Khan Assir, Ali Jawa, Hafiz Ijaz Ahmed

**Affiliations:** 1 Resident Department of Medicine, Allama Iqbal Medical College/Jinnah hospital Lahore, Medical Unit 1, Jinnah hospital Lahore, Allama Shabbir Ahmed Usmani Road Lahore, 54590, Lahore, Pakistan; 2 Department of Medicine, Division of Endocrinology, Allama Iqbal Medical College/Jinnah hospital Lahore, Lahore, Pakistan; 3 Division of Nephrology, Allama Iqbal Medical College/Jinnah hospital Lahore, Lahore, Pakistan

**Keywords:** Dengue fever, Expanded dengue syndrome, Thyroiditis, Intracerebral Hemorrhage

## Abstract

**Background:**

Although most symptomatic dengue infections follow an uncomplicated course, complications and unusual manifestations are increasingly being reported due to rising disease burden. Expanded dengue syndrome is a new entity added into World Health Organization (WHO) classification system to incorporate this wide spectrum of unusual manifestations. We report a case of expanded dengue syndrome with subacute thyroiditis and intracerebral hemorrhage. This is the first case report of thyroiditis in dengue infection.

**Case presentation:**

A 20 years old man presented with fever, myalgias, arthralgias, retro-orbital pain, vomiting and gum bleeding during a large dengue outbreak in Lahore, Pakistan. On 7^th^ day of illness patient became afebrile, but he developed severe headaches, unconsciousness followed by altered behavior. On 9^th^ day of illness patient developed painful neck swelling accompanied by fever, tremors, palpitations, hoarseness of voice and odynophagia. Examination revealed acutely swollen, tender thyroid gland along with features of hyperthyroidism. Laboratory evaluation revealed stable hematocrit, thrombocytopenia and leukopenia. Patient had seroconverted for anti-dengue IgM antibodies on the 10^th^ day of illness. A non-contrast Computed Tomogram (CT) of the brain showed right frontal lobe hematoma. Thyroid profile showed increased free T3 and T4 and low TSH. Technetium thyroid scan showed reduced tracer uptake. He was diagnosed as having subacute thyroiditis and treated with oral prednisolone and propranolol. Follow up CT brain showed resolving hematoma. Patient’s recovery was uneventful.

**Conclusion:**

Subacute thyroiditis may develop during the course of dengue fever and should be included as a manifestation of expanded dengue syndrome. It should be suspected in patients with dengue fever who develop painful thyroid swelling and clinical features of hyperthyroidism.

## Background

Infection with dengue virus is asymptomatic in majority of the cases. Based on 1997 World Health Organization (WHO) classification, symptomatic dengue infection may be in the form of dengue fever (DF), dengue hemorrhagic fever (DHF) and dengue shock syndrome (DSS) [1]. In 2009 WHO classification system, dengue fever was divided into dengue with or without warning signs and severe dengue [2]. However in 2011 revised WHO guidelines, dengue was divided into dengue fever (DF), dengue hemorrhagic fever (DHF) without shock or with shock (DSS) and expanded dengue syndrome [3].

Expanded dengue syndrome is a new entity added to the classification system to incorporate a wide spectrum of unusual manifestations of dengue infection affecting various organ systems including gastrointestinal, hepatic, neurological [4], pulmonary and renal systems. Dengue is the most rapidly spreading mosquito borne viral disease in the world [2] and as larger proportion of population is being affected, more unusual manifestations are being reported. We report a case of expanded dengue syndrome with subacute thyroiditis and intracerebral hemorrhage. To the best of our knowledge, this is the first case report of subacute thyroiditis in a patient with dengue fever.

## Case presentation

A 20 years old man was referred to tertiary care hospital for evaluation of altered behavior and fever during a large epidemic of dengue fever in Punjab in 2011. Patient had high grade fever for which he took self-prescribed medicines including acetaminophen and ibuprofen. Fever was accompanied by myalgias, arthralgias, headache, retro-orbital pain, rigors, chills, vomiting and gum bleeding. On 7^th^ day of illness his fever resolved, but he developed severe headache and within few hours became unconscious. Patient regained consciousness after two hours but headache, altered behavior and loss of bladder control persisted. In this condition (ie 8^th^ day of illness) he was brought to the hospital. These symptoms improved over the next 36 hours. On second day of hospitalization patient complained of sore throat and painful swelling in front of neck accompanied by high grade fever, dysphagia, hoarseness of voice, palpitations, tremors and increased frequency of bowel movement.

Two of his other family members also had fever and were diagnosed as having dengue fever.

Physical examination was remarkable for tachycardia (pulse 110/min), fever (102.6°F), hoarse low intensity voice, fine tremors and thyroid swelling. Thyroid was swollen, warm and tender and patient had painful deglutition. There was no cervical lymphadenopathy. Fundoscopy revealed a small hemorrhage over nasal aspect of right optic disc but no papilloedema. All cranial nerves were intact and there was no motor or sensory deficit.

**Figure 1:**
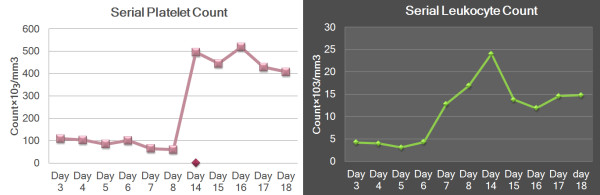
**Patterns of platelet and white cell counts during the illness.** Left panel shows platelet counts during the course of the illness. There is initial thrombocytopenia followed by thrombocytosis and subsequent normalization of platelet count. Transient thrombocytosis is probably a response to acute inflammation of thyroid. Right panel shows white cell count. Initially there is progressive leucopenia followed by leukocytosis at the time of thyroiditis.

**Figure 2:**
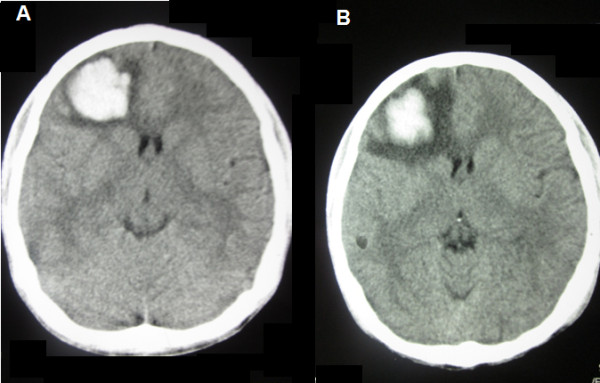
**Non-Contrast Computed Tomogram of Brain.A**.) CT brain obtained at day 8 of the illness showing a right frontal lobe hematoma seen as a hyperdense lesion surrounded by a thin rim of hypodense area of edema. There is no midline shift. **B**.). Follow up CT brain obtained on 14^th^ day of illness shows resolving hematoma. Size of hematoma has decreased, however surrounding cerebral edema has increased.

His serial complete blood counts showed stable hematocrit (range 41.2% to 44%), progressively decreasing platelet count (109,000/mm^3^ on day 4 to 60,000/mm^3^ on day 8) and leukocyte count (4,200/mm^3^ on day 4 to 3,100/mm^3^ on day 7). There was a sudden rise in leukocyte count from 3,100/mm^3^ on day 7 to 12,800/mm^3^ on day 8 and 24,000/mm^3^ on day 10, coinciding with onset of thyroid swelling (Figure 1). Platelet count started improving after day 8 of illness. Serum AST levels were 117 U/L and 67 U/L at admission and discharge respectively. Serum ALT levels were 62 U/L and 40 U/L at admission and discharge respectively. During febrile phase anti-dengue IgM ang IgG were tested negative on screening test (lateral flow immunoassay). Anti-dengue IgM by ELISA was negative on day 3 and became positive on day 10. Anti-dengue IgM antibodies were measured by indirect IgM ELISA using commercially available kit (Human GmbH, Wiesbaden, Germany). The ELISA for indirect IgM antibody detection uses Dengue specific antigens (DEN-Ag) coated on microtiter wells. IgM titre on day 10 was 1.937 (cutoff value 0.524). Non-contrast Computed Tomography of brain showed a hyperdense area in right frontal lobe surrounded by a ring of hypodensity (Figure 2). These findings were consistent with the diagnosis of right frontal lobe hematoma with surrounding edema. Ultrasonography of neck revealed diffuse heterogeneity and low intensity vascular flow in the thyroid area without evidence of abscess formation. Thyroid function tests showed markedly high free T3 (270.9 ng/dl; reference range 75-195 ng/dl) and T4 (724.0 nM/L; reference range 60-140 nM/L), and low Thyroid Stimulating Hormone (TSH) (0.01 mU/L; reference range 0.5-5 mU/L). Erythrocyte sedimentation rate (ESR) by Westergren’s method was 62 mm in 1^st^ hour. On Technetium scanning of the thyroid, the thyroid gland was not visualized due to poor tracer uptake (0.1%; reference range 1-4%).

Patient had no evidence of plasma leakage in the form of ascites, pleural effusion or hypoalbuminemia.

Fever was treated with acetaminophen and tepid sponging. Raised intracranial pressure due to intracerebral hemorrhage was treated with head elevation and intravenous mannitol. Thyroiditis was treated initially with 40 mg daily dose of oral prednisolone and pain improved in two days. Dose was subsequently reduced to 10 mg daily and was tapered off in two weeks. Oral propranolol was given for relief of symptoms related to hyperthyroidism during the first week. Patient was discharged on 8^th^ post-admission day.

## Discussion

A large outbreak of dengue fever hit Pakistan in 2011 and more than a million people suffered from symptomatic dengue infection. As a result a large number of cases with atypical manifestations of the disease were seen during this epidemic. Patient described in this case report is one example and reinforces the concept of classifying these atypical presentations as separate entity called expanded dengue syndrome [3].

Our patient had two atypical manifestations of dengue infection: intracerebral hemorrhage and subacute thyroiditis. Intracranial hemorrhage is a rare but well recognized complication of dengue fever [5,6]. These patients can present with severe headache, loss of consciousness, localizing signs or altered behavior depending upon site and size of bleeding. Our patient had altered behavior and loss of bladder control that is characteristically seen in frontal lobe lesions. Intracerebral hemorrhage should be suspected in patients with dengue fever who develop headache and/or change in mental status. Hemorrhage within the tumor of pituitary gland resulting in pituitary apoplexy has also been reported during dengue infection [7]. Our patient responded well to supportive therapy directed at lowering the raised intracranial pressure and showed rapid improvement over 48 hours without any residual neurological deficit.

Subacute thyroiditis is characterized by neck pain or discomfort, a tender diffuse goiter, and a predictable course of thyroid function evolution. Hyperthyroidism is typically the presentation followed by euthyroidism, hypothyroidism, and ultimately restoration of normal thyroid function. Subacute thyroiditis is presumed to be caused by a viral infection or a postviral inflammatory process. The majority of patients have a history of an upper respiratory infection prior to the onset of thyroiditis (typically two to eight weeks beforehand). Clusters of cases have been reported in association with Coxsackievirus, mumps, measles, adenovirus, and other viral infections [8]. Approximately one-half of patients have symptoms and signs of hyperthyroidism [9]. There are no trials assessing optimal treatment of subacute thyroiditis. Patients are treated with acetylsalicylic acid (aspirin), a non-steroidal anti-inflammatory drug (e.g. Ibuprofen) or prednisolone [10]. Therapy for hyperthyroidism is often not needed as symptoms, if present, are mild and short-lived. Those few patients who have bothersome symptoms of hyperthyroidism, may benefit from treatment with a beta blocker.

Subacute thyroiditis has not previously been reported in association with dengue virus infection. Due to high bleeding risk associated with use of aspirin or an NSAID in the setting of thrombocytopenia and frontal lobe hematoma, we preferred the use of oral prednisolone to treat the acute inflammation in our patient.

## Conclusion

Subacute thyroiditis may develop during the course of dengue fever and should be included as a manifestation of expanded dengue syndrome. It should be suspected in patients with dengue fever who develop painful thyroid swelling and clinical features of hyperthyroidism.

## Consent

Written informed consent was obtained from the patient for publication of this Case report and all accompanying images. A copy of the written consent is available for review by the Series Editor of this journal.

## Abbreviations

DF: Dengue Fever; DHF: Dengue Hemorrhagic Fever; DSS: Dengue Shock syndrome; ELISA: Enzyme Linked Immunosorbent Assay; fT3: Free Tri-iodo-thyronine; fT4: Free Thyroxine; TSH: Thyroid Stimulating Hormone; NSAID: Non-Steroidal Anti-Inflammatory Drug.

## Competing interests

The authors declare that they have no competing interests.

## Authors’ contributions

MZKA carried out the literature search and drafted the manuscript; AJ and HIA did the critical revision for important intellectual content in the manuscript. All authors read and approved the final manuscript.

## Pre-publication history

The pre-publication history for this paper can be accessed here:

http://www.biomedcentral.com/1471-2334/12/240/prepub
